# Drinking and driving relapse: Data from BAC and MMPI-2

**DOI:** 10.1371/journal.pone.0209116

**Published:** 2019-01-02

**Authors:** Paolo Roma, Cristina Mazza, Giorgia Ferracuti, Maria Elena Cinti, Stefano Ferracuti, Franco Burla

**Affiliations:** Department of Human Neuroscience, Faculty of Medicine and Dentistry, Sapienza University of Rome, Rome, Italy; Universita degli Studi Europea di Roma, ITALY

## Abstract

Road traffic injuries are the ninth cause of death across all age groups, globally (WHO, 2015). Many road traffic crashes are caused by Driving Under the Influence (DUI) of alcohol by persons who have previously had their license suspended for the same reason. The aim of this study was to identify specific risk factors and personality characteristics in repeat offenders. The sample was comprised of 260 subjects who were not repeat DUI offenders (DUI-NR), but had a single license suspension between 2010 and 2011; and 97 repeat offenders who received at least two DUI convictions within a period of 5 years. At the time of their first driving license suspension, participants provided their blood alcohol content (BAC) and completed a valid MMPI-2 test. ANOVA and MANOVAs were performed to determine whether there were significant differences in BAC and MMPI-2 profiles between DUI-NR and DUI-R participants and a logistic regression was run to identify whether BAC at the time of the first suspension and specific personality features could predict recidivism. A two-step cluster analysis was run to identify recidivist typologies. Results showed that, relative to DUI-NR participants, DUI-R participants had higher BAC at the time of their first conviction and more problematic MMPI-2 profiles, despite the presence of social desirability responding. The best predictors of recidivism were BAC and the scales of Lie (L), Correction (K), Psychopathic Deviate (4-Pd), Hypomania (9-Ma), and Low Self-Esteem (LSE). Two-step cluster analyses identified two recidivist profiles, according to 32 selected MMPI-2 validity, clinical, content, supplementary, and PSY-5 scales. Comparisons with previous research are discussed and ideas for further study are generated.

## Introduction

Drinking and driving, despite being harmful, is a widespread behavior with a significant impact on social policies. According to the Global Status Report on Road Safety of the World Health Organization [1], “over 1.2 million people die each year on the world’s roads, with millions more sustaining serious injuries and living with long-term adverse health consequences. Globally, road traffic crashes are the main cause of death among those aged 15–29 years, while road traffic injuries are currently estimated to be the ninth leading cause of death across all age groups globally and are predicted to become the seventh leading cause of death by 2030” (p. X). Beirness and Davies (2007) [2], in their study conducted in Canada using anonymous telephone interviews, showed that 11.6% of the population with a driving license (men: 78.1%) drive within 1 hour of consuming two or more alcoholic drinks. In Italy, the percentage of road traffic deaths involving alcohol is 25% [1]; in 2014, the most recent year for which data are available, there were 44,566 subjects found *driving under the influence of alcohol (DUI)*, of whom 2,400 were younger than 21 years, new drivers, or professional drivers. In 2016, law n.41 officially introduced the crimes of road killing and causing personal road injuries to Italy.

Most people caught drinking and driving, after receiving a harsh punishment, drive again under the influence of alcohol after regaining their license [3–5]. DUI offenders’ recidivism is estimated to fall in the range of 21–47% [6–7]; however, this estimate is largely conservative, considering that it does not include subjects who drink and drive without being re-arrested. Previous studies have attempted to predict recidivism using different sets of variables; however, they have not shown agreement [8–9]. For example, Impinen et al. (2009) [10] found that recidivists are, on average, younger and have higher *blood alcohol concentration (BAC)* than non-recidivists at the time of their first offense. Fitts, Palk, Lennon, and Clough (2017) [11] reported that subjects with a very high number of BAC convictions are more likely to re-offend, confirming the results of Chou et al. (2005) [12] and Morrison, Begg, and Langley (2002) [13]. Others have attempted to predict recidivism using a combination of demographic variables and personality characteristics, employing statistical procedures such as multiple regression, discriminant function analysis, and logistic regression (for a review, see Nochajski & Stasiewicz, 2006 [14]). From these studies, we focused on those that drew on BAC and the *Minnesota Multiphasic Personality Inventory-2 (MMPI-2)* [15]. The MMPI-2 is the most widely used psychodiagnostic tool; it is used in multiple contexts [16], including the driving license consultation following a DUI suspension [17].

Craig and Dres (1989) [18] compared BAC and the MMPI profiles of 100 DUI recidivist subjects and 100 non-recidivist subjects in order to identify significant differences between the groups that could predict recidivist behavior. The authors considered 28 MMPI scales (4 validity scales, 10 basic scales, and 14 content scales) and found that recidivists scored higher on the scales of Frequency (F), Psychopathic Deviate (4-Pd), Social Introversion (0-Si), Health Concerns (HEA), and MacAndrew Alcoholism-Revised (MAC-R). The set of variables predicted 10% of the variance. C’de Baca, Miller, and Lapham (2001) [19] administered a medical history form, the Alcohol Use Inventory, and the MMPI-2 to 1,496 subjects. From the measures, they selected five scales for in-depth analysis: Anger (ANG), Antisocial Practices (ASP), Depression (2-D), Low Self Esteem (LSE), and MAC-R. They identified five risk factors that could be used to identify offenders at high risk for DUI recidivism: a) age (younger than 29 years); b) years of education (<12 years); c) BAC at the time of arrest (.20 or greater); d) score on the Receptive Area of the Alcohol Use Inventory (raw score of 7 or greater); and e) score on the MAC-R (raw score of 23 or greater). Cavaiola, Strohmetz, and Abreo (2007) [20] performed a longitudinal follow-up study evaluating the profile of 77 subjects who were tested 12 years before an evaluation of fitness to drive. Among these, 38% (29 subjects) were recidivists. For these respondents, scores on the MMPI-2 validity scales of Lie (L) and Correction (K), showing symptom minimization, were significantly higher than those of non-recidivists. Finally, Shim, Wang, and Bahk (2016) [21], administering the MMPI-2 validity and clinical scales, found higher scores on the F and 2-D scales in 80 individuals with multiple DUI offenses compared to individuals with a single DUI offense. The authors also performed a hierarchical cluster analysis, identifying two groups of multiple offenders: one group characterized by relatively high scores on the 2-D and 4-Pd scales; and another (larger) group with lower scores on the Hypomania (9-Ma) and 0-Si scales, as well as a V-shaped profile on the validity scales. Globally, these studies underline significant but non-stable differences in some MMPI-2 scales among DUI single (or first) offenders and DUI recidivists. Nevertheless, the studies cited above differ from each other in the number of MMPI-2 scales considered and the width of the examined sample. Accordingly, our main aim was to improve knowledge in the field of DUI research, using 32 MMPI-2 scales to compare one of the largest groups of DUI recidivists (DUI-R) with DUI single (or first) offenders (DUI-NR).

We were interested in examining whether any MMPI-2 variables could be used to identify subjects likely to become recidivists. Such identification could be used at the time of a first conviction to instigate tailored treatment with a specific focus on features that could prevent the risk of relapse.

Specifically, following the results of the quoted research, we hypothesized:

H1: BAC at the time of the first offence would be higher in recidivists (DUI-R) compared to single DUI offenders (DUI-NR).

H2: Relative to DUI-NR subjects, DUI-R subjects would obtain higher values on the MMPI-2’s two principal validity scales of symptom minimization (L and K) and higher values on the 2-D, 4-Pd, 0-Si, MAC-R, and LSE scales.

H3: Building on the study of Shim et al. (2016) [21], which used the MMPI-2 to cluster DUI multiple offenders, we wondered if it would be possible to also use MMPI-2 selected content, supplementary, and PSY-5 scales to more deeply classify DUI-R subjects.

H4: Finally, to determine whether there are specific profiles indicating a risk and danger of recidivism, we investigated whether any identified differences between DUI-NR and DUI-R groups could be useful in predicting DUI recidivism.

## Materials and methods

### Participants

Among the 428 DUI Caucasian subjects who registered a negative drug test at the time of their conviction, had only a single license suspension, and completed the MMPI-2 in our laboratory between January and December 2011 (to assess whether they met the psychiatric and psychological requirements for safe driving), the following subjects were excluded: a) the few female subjects (*N* = 13), in order to prevent confounding gender variables; b) subjects who had not been born and raised in Italy (*N* = 21), in order to avoid linguistic or cultural biases; c) the very few subjects whose police report did not include a BAC measurement (*N* = 4); d) subjects who claimed to have used drugs in the past (*N* = 19); and e) subjects who did not provide informed consent to anonymous data processing for the purpose of research (*N* = 14). The final sample was comprised of 357 male subjects. All subjects were residents of the Lazio region and were sent to our laboratory in Rome by medical-legal commissions throughout the region.

In 2017, a case file from the medical-legal Commission of Motorization indicated that, among the 357 subjects evaluated in 2011, there were: 97 DUI recidivists (subjects who had received an additional driving license suspension after 2011 during a verification check or at a roadblock); and 260 non-repeat DUI offenders (subjects who had passed all medical controls, even 5 years after re-attaining their driving license). Complete demographic characteristics are shown in Table 1. No statistically significant differences were observed across categories in age, years of education, or marital status. This study was carried out with written informed consent by all subjects and was approved by the local ethics committee (Board of the Department of Human Neuroscience, Faculty of Medicine and Dentistry, Sapienza University of Rome).

**Table 1:** Descriptive statistics of the sample.

		DUI-NR *N* = 260	DUI-R *N* = 97	Total Sample *N* = 357
**Age**_**years**_	*M (SD)*	36.57 (11)	38.04 (9.7)	36.97 (10.7)
	*Min–Max*	20–75	20–75	20–75
**Education**	*M (SD)*	12.79 (4.2)	12.9 (3.8)	12.82 (4.1)
	*Min–Max*	8–18	8–18	8–18
**Marital Status**	*% single*	32	35	34.2
	*% married*	42.3	48.1	46.5
	*% divorced*	25.8	16.9	19.3

## Measures

### Blood alcohol concentration

Blood alcohol concentration (BAC), also called blood alcohol content, blood ethanol concentration, or blood alcohol level, is commonly used as a metric of alcohol intoxication for legal or medical purposes. For the present study, BAC data were gathered from the DUI police reports at the time of the first driving license suspension. The current Italian legislation sets the maximum BAC level for driving at 0.5 g/mL.

### MMPI-2

The full version of the MMPI-2 [15] was administered individually to all participants, according to standard instructions. The Italian version of the inventory consists of 567 items [22]. In line with the advice given in the technical manual [15], we excluded protocols with a Cannot Say Scale > 30 or a VRIN or TRIN T score > 80. We did not make exclusions based on findings from other validity scales, ensuring a full range of validity scale scores for analysis (nevertheless, no exclusion would have been made according to the rules of the technical manual). According to the clinical meaning of the scales, previous studies using the MMPI and MMPI-2, and other studies of personality characteristics related to drinking and driving behavior [23–27] and recidivist alcohol consumption in DUIs [28–29], we selected from the MMPI-2: a) 3 principal validity scales (F, L, K); b) 9 (out of 10) standard clinical scales (excluding the Masculinity–Femininity [MF] scale); c) 14 content scales; d) 4 supplementary scales; and e) 2 Personality Psychopathology Five [PSY-5] scales. Scale scores were calculated in standard T points (*M* = 50, *SD* = 10), which is the traditional method of measuring the MMPI-2 [30], with K correction for the 1-Hs, 4-Pd, 7-Pt, 8-Sc, and 9-Ma scales. The T point classification was: 55–60: moderately high; 60–65: high; and 65–70: very high [15]. As the tests were administered in 2011, the normative samples used to compute the row T point scores were: the sample that had been in use since 1995 [31] and the 2011 update of this sample [32].

## Statistical analysis

The first purpose of this study was to test differences between BAC and the MMPI-2 scores of the DUI-NR and DUI-R groups. Accordingly, a univariate analysis of variance (ANOVA) test was run to detect BAC differences between these groups. Secondly, a multivariate analysis of variance (MANOVA) test was run using the two attitudes (single offence vs. recidivism) as independent variables and MMPI-2 scale T points as dependent measures. We inspected the effect sizes of the score differences between groups. A two-step cluster analysis with the BIC criterion was used to define the profiles of multiple offenders. This method first identified groupings using a quick cluster algorithm (pre-clustering) and then ran hierarchical cluster models in the second step. MMPI-2 scales were used in the cluster model. In order to achieve natural clustering, the number of clusters was set to automatic. Finally, one of the questions addressed in this study was whether there are any indicators at the time of the first DUI arrest that might identify individuals who are more likely to become repeat offenders. In order to answer this question, we ran a logistic regression using variables found to differ between groups (DUI-NR and DUI-R) in the previous ANOVA as predictors.

Information on the distributive properties (skewness and kurtosis) of all analytical variables is provided in S1 File. All analyses were performed with SPSS for Windows, version 22.0.

## Results

### BAC

The ANOVA showed a significant difference between the DUI-NR and DUI-R groups in BAC at the time of the first driving license suspension (see Table 2). It is interesting to note that the mean BAC of the DUI-R group was almost three times the maximum allowed by current Italian legislation (0.50 g/mL), demonstrating the severity of the social problem.

**Table 2:** Mean differences in BAC Between DUI-NR and DUI-R at first driving license suspension.

BAC_g/mL_	DUI-NR *N* = 260	DUI-R *N* = 97	Total Sample *N* = 357
*M (SD)*	.98 (.33)*	1.29 (.30)*	1.06 (.35)
*Min–Max*	.38–1.78	.68–1.94	.38–1.94

*p < .001.

### MMPI-2 scales

A 2 x 32 MANOVA (groups x selected MMPI-2 scales) showed a significant attitude effect (single offense vs. recidivism) on the MMPI-2 scales, *V* = 0.84, *F*(32, 324) = 54.44, *p* < .001, parη^2^ = .843. We applied the Bonferroni correction for multiple comparisons. Separate univariate ANOVAs on the outcome variables revealed a significant attitude effect on: a) all validity scales (L, F, and K); b) all clinical scales except 3-Hy [*F*(1, 355) = 2.09, *p* = .149, parη^2^ = .006]; c) all content scales save the FRS [*F*(1, 355) = .76, *p* = .383, parη^2^ = .002], OBS [*F*(1, 355) = 3.21, *p* = .074, parη^2^ = .009], ANG [*F*(1, 355) = .44, *p* = .508, parη^2^ = .001], and TPA [*F*(1, 355) = .99, *p* = .321, parη^2^ = .003]; and d) two supplementary scales (HO and MAC-R). However, no effect was found on the O-H [*F*(1, 355) = .16, *p* = .703, parη^2^ = .000] and R scales [*F*(1, 355) = .34, *p* = .558, parη^2^ = .001], or the PSY-5 scales DISC [*F*(1, 355) = 2.36, *p* = .126, parη^2^ = .007] and AGGR [*F*(1, 355) = .62, *p* = .433, parη^2^ = .002]. Table 3 shows the descriptive values of the two groups (DUI-NR vs. DUI-R) for all significant outcome variables. Compared to the DUI-NR group, the DUI-R group scored higher on all MMPI-2 scales, despite the fact that their profiles contained more underreporting [L and K > 60 and F < 55]. Overall, DUI-R respondents showed profiles more worthy of clinical attention, with moderately high elevation on the 6-Pa, 9-Ma, and LSE scales and high scores on the 4-Pd and MAC-R scales, in addition to their aforementioned high scores on the L and K scales.

**Table 3:** Statistically significant differences in MMPI-2 scales between DUI-NR and DUI-R.

	MMPI-2	DUI-NR *N* = 260 *M* (DS)	DUI-R *N* = 97 *M* (DS)	*F*	parη^2^
**Validity** **Scales**	L	57.98 (9.24)	62.12 (11.18)	12.62***	.034
	F	48.45 (9.71)	54.89 (8.7)	32.74***	.084
	K	55.91 (8.12)	61.05 (9.47)	25.82***	.068
**Clinical** **Scales**	1-Hs	45.61 (4.91)	49.59 (7.58)	33.66***	.087
	2-D	48.65 (7.52)	51.42 (8.42)	8.96**	.025
	4-Pd	52.65 (7.03)	62.77 (6.5)	152.53***	.301
	6-Pa	50.34 (8.94)	55.33 (9.64)	21.06***	.056
	7-Pt	50.68 (8.49)	53.79 (9.26)	9.03**	.025
	8-Sc	47.87 (8.79)	51.18 (11.31)	8.5**	.023
	9-Ma	51.89 (9.66)	55.93 (11.52)	11.07***	.030
	0-Si	46.25 (6.93)	48.45 (8.31)	6.41*	.018
**Content** **Scales**	ANX	47.66 (7.23)	50.39 (8.85)	8.87**	.024
	DEP	45.22 (7.19)	49.27 (10.18)	17.65***	.047
	HEA	50.83 (7.74)	53.98 (9.72)	10.09**	.028
	BIZ	48.01 (7.48)	50.96 (8.3)	10.32***	.028
	CYN	48.11 (9.24)	51.44 (10.57)	8.48**	.023
	ASP	48.50 (10.28)	51.57 (9.37)	6.61*	.018
	LSE	44.92 (6.71)	56.36 (7.96)	185.16***	.343
	SOD	46.30 (7.13)	48.48 (8.2)	6.08*	.017
	FAM	46.00 (6.86)	49.09 (8.79)	12.22***	.033
	WRK	45.20 (6.24)	47.99 (7.8)	12.23***	.033
**Supplementary** **Scales**	HO	47.5 (9.49)	50.59 (10.36)	7.11**	.020
	MAC-R	56.30 (7.13)	61.35 (8.46)	31.87***	.082

*p ≤.05.

**p ≤.01.

***p ≤.001.

### Cluster analysis

The two-step cluster analysis of the 97 subjects with multiple offenses revealed two clusters with significant differences in mean score profiles (see Table 4). A 2 x 32 MANOVA showed a significant clustering effect (cluster 1 vs. cluster 2) on the MMPI-2 scales, *V* = 0.76, *F*(32, 64) = 6.45, *p* < .001, parη^2^ = .763. In more detail, separate univariate ANOVAs on the outcome variables revealed a significant clustering effect on all selected scales, except on the L [*F*(1, 95) = 2.76, *p* = .101, parη^2^ = .028], 3-Hy [*F*(1, 95) = .05, *p* = .821, parη^2^ = .001], 4-Pd [*F*(1, 95) = 3.31, *p* = .072, parη^2^ = .034], 8-Sc [*F*(1, 95) = .08, *p* = .776, parη^2^ = .001], and 9-Ma [*F*(1, 95) = .067, *p* = .797, parη^2^ = .001] scales. Characteristics of the DUI-R respondents in each cluster are summarized as follows. Recidivists in cluster 1 had higher scores on all MMPI-2 scales compared to recidivists in cluster 2, save for the K, R, and O-H scales.

- Cluster 1 (*N* = 51): Recidivists had high scores (T points ≥ 60) on the LSE and MAC-R scales and moderately high scores on the F validity scales; the 7-Pt clinical scale; the ANX, BIZ, DEP, ASP, CYN, HEA, and FRS content scales; the HO supplementary scale; and the DISC PSY-5 scale. All other MMPI-2 scales showed T points < 55.
- Cluster 2 (*N* = 46): Recidivists had high scores (T points ≥ 60) on the K scale and moderately high (> 55 T points) to high (> 60 T points) scores on the R scale.

**Table 4:** Mean differences in MMPI-2 selected scales between cluster 1 and cluster 2 in DUI-R.

	MMPI-2	Cluster 1 *N* = 51 *M* (DS)	Cluster 2 *N* = 46 *M* (DS)	F	parη^2^
**Validity** **Scales**	L	60.35 (10.65)	64.09 (11.55)	2.74	.028
	F	56.80 (8.77)	52.76 (8.18)	5.47*	.054
	K	57.90 (10.30)	64.54 (7.05)	13.43***	.124
**Clinical Scales**	1-Hs	53.00 (7.96)	45.80 (9.95)	27.88***	.227
	2-D	53.37 (9.99)	49.26 (5.58)	6.07*	.060
	3-Hy	51.39 (11.44)	51.83 (6.38)	.05	.001
	4-Pd	61.65 (6.08)	64.02 (6.78)	3.31	.034
	6-Pa	58.63 (9.21)	51.67(8.82)	14.34***	.131
	7-Pt	58.53 (9.51)	48.54 (5.38)	39.34***	.293
	8-Sc	50.86 (11.12)	51.52 (10.46)	.08	.001
	9-Ma	56.22 (12.61)	55.61 (10.30)	.07	.001
	0-Si	52.49 (8.24)	43.98 (5.73)	34.16***	.264
**Content** **Scales**	ANX	55.90 (7.17)	44.28 (6.11)	72.96***	.434
	FRS	55.27 (8.52)	47.41 (7.50)	23.04***	.195
	OBS	53.14 (8.30)	41.87 (5.61)	60.01***	.387
	DEP	55.22 (10.10)	42.67 (4.84)	58.81***	.382
	HEA	58.22 (6.66)	49.28 (7.93)	25.71***	.213
	BIZ	53.02 (6.92)	45.35 (5.70)	67.80***	.416
	ANG	50.06 (9.84)	41.93 (5.26)	24.92***	.208
	CYN	57.51 (10.41)	44.72 (5.47)	55.55***	.369
	ASP	56.98 (9.15)	45.57 (4.91)	56.83***	.374
	TPA	49.22 (10.21)	41.15 (5.65)	22.48***	.191
	LSE	60.27 (7.24)	52.02 (6.37)	35.19***	.270
	SOD	51.90 (8.78)	44.70 (5.45)	22.98***	.195
	FAM	54.92 (7.14)	42.63 (5.37)	92.35***	.493
	WRK	52.86 (6.72)	42.59 (4.79)	73.76***	.437
**Supplementary** **Scales**	MAC-R	64.90 (8.78)	57.41 (6.05)	23.41***	.198
	OH	44.29 (3.91)	47.91 (2.70)	27.60***	.225
	HO	57.35 (8.79)	43.09 (5.80)	86.99***	.478
	R	50.59 (10.61)	57.67 (9.16)	12.26***	.114
**PSY-5** **Scales**	DISC	55.61 (10.06)	49.24 (7.41)	12.37***	.115
	AGGR	53.08 (12.04)	47.09 (6.76)	8.87**	.085

*p ≤.05.

**p ≤.01.

***p ≤.001.

### Regression analysis

A multiple logistic regression analysis was performed using the stepwise method. DUI recidivism was set as the dependent variable while BAC and the MMPI-2 scales were used as covariates. The inserted variables were those that showed a statistical difference in the previous multivariate ANOVA comparing the DUI-NR and DUI-R groups. We selected the MMPI-2 scales with scores in the moderately high range (> 55 T points) or higher, according to the standard classification [15]. Specifically, these included the L, K, 4-Pd, 6-Pa, 9-Ma, LSE, and MAC-R scales (see Table 5).

**Table 5:** Logistic regression model predicting recidivisim.

	T	E.S.	Wald	gl	Sign.	Exp(B)
**BAC**	3.291	.702	21.956	1	≤.001	26.882
**L**	.057	.024	5.574	1	.018	1.059
**K**	.085	.031	7.620	1	.006	1.089
**4-Pd**	.198	.031	41.233	1	.≤001	1.219
**9-Ma**	.042	.021	4.172	1	.041	1.043
**LSE**	.234	.035	45.382	1	≤.001	1.264
**Costant**	-40.400	5.023	64.699	1	≤.001	.000

The Wald test was used to evaluate the contribution of each individual predictor to the model. A predictor was entered into the regression equation when the probability (*p*) was 0.05. Overall, prediction success was 91.3% (95.4% for DUI-NR and 80.4% for DUI-R). The prediction model showed goodness of fit to our observed data (χ^2^ [7] = 272.19, *p* < .000). Nagelkerke’s R of .774 indicated a moderately strong relationship between prediction and grouping. The final prediction model was comprised of the BAC, L, K, 4-Pd, 9-Ma, and LSE scales. 6-Pa and MAC-R were excluded (see Table 6).

**Table 6:** Covariates not included in the prediction model.

	Score	gl	Sign.	
**6-Pa scale**	2.059	1	.151	
**MACR**	3.803	1	.051	
**Global Statistics**	4.870	2	.088	

## Discussion

The main aim of our study was to investigate which variables could help clinicians detect DUI offenders at risk of relapse. To address this topic, we analyzed subjects’ BAC at the time of their first conviction and their MMPI-2 profiles—measures that experts have easy access to and with which they can work.

With respect to BAC, previous studies have found higher BAC at the time of the first DUI offence in recidivist subjects [33–36]. Our data are aligned with these previous findings, confirming the hypothesis (H1) that DUI-R respondents’ BAC was higher than that of the DUI-NR respondents. It is interesting to note that the mean BAC found in the present study (1.20 g/mL) is higher than that found in other studies. Future studies could identify the best value (cut-off) of BAC at the first screening to reliably discriminate subjects at high risk of recidivism.

Our data also confirmed the second hypothesis (H2), showing that recidivists obtained higher scores on MMPI-2 scales measuring symptom minimization, depression, psychopathic deviance, social attitude, and alcoholic personality. More specifically, the K and L scores of the DUI-R group suggested a higher level of self-favorable bias relative to the DUI-NR group, with unrealistic self-reported adjustment, a need to deny problems and psychological weaknesses, and a tendency to present an image of adequacy and self-control that is inconsistent with real life. DUI-R subjects seemed to be more prone to lie about their personality characteristics, wanting to appear free of psychological defects and more socially adapted than non-recidivists. Similar results were found by Cavaiola, Strohmetz, and Abreo (2007) [20]. Furthermore, in the present study, the higher scores on the 2-D scale found in the DUI-R group are compatible with the results of Shim et al. (2016) [21], who found major depression in the group of repeat offenders. The relation between depression and alcohol addiction is well established in the literature [37]. Moreover, in our sample—and as also found by Craig and Dress in 1989 [18]—recidivists had higher scores on the 4-Pd and 0-Si scales. Their profiles showed a lack of concern about most social and moral standards of conduct, rebellion towards authority figures, and engagement in antisocial acts. These subjects had histories of underachievement, were impulsive, and strove for immediate gratification of impulses with a limited frustration tolerance. Furthermore, they did not plan their behavior well, tended to act without considering the consequences of their actions, and generally did not learn from their experience. As expected, we also found higher scores on the MAC-R scale in the DUI-R group, in line with the findings of previous research [18] [19] [38]. However, this result was not found in studies conducted by Cavaiola [20] [39] or Shim et al. (2016) [21]. Finally, relative to DUI-NR respondents, DUI-R respondents showed lower self-esteem, a lack of interest, and a sense of inefficiency and inadequacy (LSE scale).

The rest of the DUI-R profile showed a tendency to perceive discomfort, which they mainly attributed to a physical, bodily origin. Somatic stress conversion was present (F, 1-Hs). Relative to DUI-NR respondents, DUI-R respondents tended to be more sensitive to what others thought of them and suspicious of the motives of others, often seeing the environment as demanding and unsupportive. They commonly felt angry, dissatisfied, and resentful, and blamed others for their condition—especially family members (6-Pa scale). The psychological picture of DUI-R respondents was also characterized by social alienation, a sense of feeling misunderstood, and experiences of rejection (8-Sc scale). Overall, the differences between the two groups indicated that the MMPI-2 psychological profile of recidivist subjects was more deserving of clinical attention. This profile is compatible with the results of previous studies [40].

Building on the study of Shim et al. (2016) [21], which used MMPI-2 validity and clinical scales to cluster DUI multiple offenders, we wondered if it would also be possible to classify DUI-R respondents using MMPI-2 content scales. We selected supplementary and PSY-5 scales (H3) in order to define recidivist typologies based on the psychological characteristics they were aware of or wished to communicate. We believe that this information could be a useful starting point for tailored interventions, even in an initial screening. Two-step cluster analysis showed two typical recidivist profiles. Recidivists in cluster 1 (53%) complained more of problems related to work and family and seemed to exhibit a number of the psychopathological characteristics of alcohol use disorder: hostility, anxiety, nervousness, restlessness, apprehension, difficulty maintaining attention and concentration, sleep disturbance, difficulty making decisions, lack of self-confidence, disconstraint, and a feeling of being overwhelmed by daily responsibilities. They also complained of medical symptomatology and demonstrated bizarre ideation processes. In cluster 2, which comprised 47% of the DUI-R group, subjects attempted to deny, rationalize, and limit self-disclosure, probably due to the evaluative/forensic setting [41–42]. They showed a constricted range of feeling, with limited emotional responsiveness across a wide spectrum. Thus, their profiles were underreporting. It is interesting to note that the two cluster groups did not differ in psychopathic deviate levels.

Finally, to investigate the predictive power (H4) of the possible differences between the DUI-NR and DUI-R groups (dependent variables), we ran a binary logistic regression, using BAC and the MMPI-2 scales (7 out of 32) that were statistically different between groups and had scores higher than 55 T-points as predictors. Results showed a model describing a tendency to deny negative emotions and cover a lack of self-esteem (in order to show more adapted functioning), combined with energy expressed as tension, hyperactivity, self-centeredness, and irritability. Recidivists may have also had difficulties controlling or preventing their thoughts from becoming actions. DUI-R subjects felt that they—and their behaviors—had no value. They did not think that what they did could affect reality: the responsibility for reality always sat with others and the locus of control of their actions was always external. Low self-esteem, combined with impulsivity and a lack of hesitation, comprised a unique and very powerful measure of a possible attitude of DUI recidivists.

## Conclusion

Our results show that DUI-R subjects are characterized by higher BAC and higher scores on many MMPI-2 scales, even if their largest tendency is to falsify personal characteristics (as shown by their scores on the validity scales). Moreover, we found that BAC at the time of first conviction and five scales of the MMPI-2 are capable of predicting recidivist behavior. Finally, among DUI-R respondents, we identified two clusters of recidivists: one comprised of subjects with a defense tendency and another showing a more problematic pattern. Our findings are clinically useful because they enable us to reliably identify two typologies of DUI subjects at high risk of relapse at the first screening phase, according to specific personality characteristics and BAC. The ability to identify early offenders at high risk for a second DUI offense allows us to impose strict medical-legal controls in order to direct preventative treatment interventions to this targeted population.

One limitation of this study is that our non-recidivist group selection criterion of 5 years without a second offence, while practical, was not completely sufficient: some individuals might have been recidivists but simply not caught in their second crime. Further limitations are that women were excluded from the study (due to the low number of female subjects in our initial screening) and all participants were residents of central Italy. Consequently, the conclusions of this study are valid only for the male population. Finally, it could be useful to evaluate subjects’ personalities through more indirect methods: although the majority of previous studies were conducted using self-administered tools, in self-administered tests the level of response counterfeiting can prevent a true understanding of subjects’ personalities and syndromes.

Our study can be a starting point for building an MMPI-2 recidivism risk index with the scales resulting in our regression model, in line with the literature on DUIs. This MMPI-2 index could be used together with BAC at the time of the first conviction. The best cut-off for BAC should be identified through future analysis of receiver operating characteristics, in order to more reliably discriminate between subjects at risk of recidivism.

The results of our study can also be useful for implementing tailored psychological treatment of subjects who, at the first withdrawal of their license, show a tendency to distance themselves from personal distress, either by running away or by acting out. The proclivity to deny emotional distress and to shift negative feelings to the motor sphere, combined with low self-esteem, should be the target of this suggested psychological treatment. Such intervention, which should be based on empirical research [43–46], should be mandatory during the assessment period before license reassignment.

## Supporting information

S1 File Skeweness and kurtosis. (XLS)
